# Supplementary material for: The Dynamic Changes of Brassica napus Seed Microbiota across the Entire Seed Life in the Field
Source: Plants (Basel). 2024 Mar 21;13(6):912. doi: 10.3390/plants13060912 (PMC10975644; doi:10.3390/plants13060912)
Supplement: Supplementary file 1 [file plants-13-00912-s001.zip › Additional file 1.pdf]

Table S1 Total numbers of reads among all samples before analysis and the number of counts after concatenation and optimization.

Groups	Sample name	Q30(%)	Reads	Counts	Goods_coverage
Flower buds	0d-Flower1	94.46	47115	36250	0.999715
	0d-Flower2	94.42	64692	51909	0.999829
	0d-Flower3	94.43	67630	58644	0.999145
	0d-Flower4	94.13	56639	47506	0.999259
	0d-Flower5	93.8	28543	21197	0.999943
	0d-Flower6	94.21	45390	35080	0.999601
	0d-Flower7	94.05	87170	75506	0.99943
Young pods	Young pod1	94.01	181140	148922	0.99943
	Young pod2	93.9	218539	159068	0.999031
	Young pod3	94.1	198094	153293	0.999145
	Young pod4	93.83	23759	19967	0.999943
	Young pod5	94.04	96417	75399	0.999373
	Young pod6	94.15	123265	92964	0.999373
	Young pod7	94.03	35372	28448	0.999772
20daf seeds	20daf-seeds1	94.82	78859	59919	0.998974
	20daf-seeds2	94.39	72858	56035	0.999259
	20daf-seeds3	94.43	80743	65745	0.998404
	20daf-seeds4	93.11	42250	20287	0.999772
	20daf-seeds5	94.23	60362	40811	0.999772
	20daf-seeds6	94.55	101093	73286	0.998746
	20daf-seeds7	94.52	51371	25531	0.999829
	20daf-seeds8	94.51	31818	25133	0.999886
30daf seeds	30daf-seeds1	94.09	52385	36452	0.999772
	30daf-seeds2	94.39	63745	55000	0.999658
	30daf-seeds3	94.25	27476	20812	0.999943
	30daf-seeds4	94.03	55310	46632	0.999772
	30daf-seeds5	94.14	30851	26816	0.999886
	30daf-seeds6	94.33	67647	52345	0.999544
40daf seeds	40daf-seeds1	94.04	183192	119256	0.999145
	40daf-seeds2	93.08	70581	35361	0.999145
	40daf-seeds3	93.71	25830	17546	0.999943
	40daf-seeds4	94.12	68650	50644	0.99886
	40daf-seeds5	94.36	40422	33420	0.999544
	40daf-seeds6	94.31	49522	39287	0.999316
	40daf-seeds7	94.47	71784	57001	0.998404
	40daf-seeds8	94.31	69520	53152	0.99886

Groups	Sample name	Q30(%)	Reads	Counts	Goods_coverage
50daf seeds	50daf-seeds1	94.55	58712	39620	0.999886
	50daf-seeds2	94.37	67725	37314	0.999715
	50daf-seeds3	94.4	30031	19878	0.999829
	50daf-seeds4	94.56	59107	46176	0.999316
	50daf-seeds5	94.37	27116	20679	1
	50daf-seeds6	94.39	45080	26888	1
	50daf-seeds7	94.39	41183	24371	0.999829
Parental seeds	Parental seeds1	91.54	66566	39082	0.996389539
	Parental seeds2	92.46	71072	57764	0.992291179
	Parental seeds3	91.08	125530	95679	0.996487119
	Parental seeds4	91.15	126545	82683	0.992096019
	Parental seeds5	91.32	36827	23405	0.99834114
	Parental seeds6	91.29	64731	41079	0.994730679
Mature seeds	Mature seeds1	91.74	35009	23241	0.99736534
	Mature seeds2	92.8	114216	77818	0.993169399
	Mature seeds3	91.63	176338	132119	0.99717018
	Mature seeds4	91.33	113115	82931	0.995608899
	Mature seeds5	91.51	127362	99381	0.995511319
	Mature seeds6	91.84	102859	69662	0.99726776
Negative control	YX1	90.32	39	9	
	YX2	87.87	125	53	
	YX3	90.06	219	131	
	YX4	87	2	0	
	YX5	87.41	121	27	
	YX6	88.28	188	47	

Table S2 The primer sequences used in this study.

PCR1-F bold sequences are the Illumina read1 sequence primer (RD1SP), non-bold sequences are the 799F primer

ACACTCTTTCCCTACACGACGCTCTTCCGATCTAACMGGATTAGATACCCKG

PCR1-R bold sequences are the Illumina read2 sequence primer (RD2SP), non-bold sequences are the1193R primer

GTGACTGGAGTTCAGACGTGTGCTCTTCCGATCTACGTCATCCCCACCTTCC

**PCR2-F**	**First half of non-bold sequences are adapters P5, bold sequences are indexes i5, second half of non-bold sequences are partial RD1SP**
1	AATGATACGGCGACCACCGAGATCTACAC**GAGCGCTA**ACACTCTTTCCCTACACGAC
2	AATGATACGGCGACCACCGAGATCTACAC**CGCTCAGT**ACACTCTTTCCCTACACGAC
3	AATGATACGGCGACCACCGAGATCTACAC**GTCTTAGG**ACACTCTTTCCCTACACGAC
4	AATGATACGGCGACCACCGAGATCTACAC**ACTGATCG**ACACTCTTTCCCTACACGAC
5	AATGATACGGCGACCACCGAGATCTACAC**AATTCTGC**ACACTCTTTCCCTACACGAC
6	AATGATACGGCGACCACCGAGATCTACAC**GGCCTCAT**ACACTCTTTCCCTACACGAC
7	AATGATACGGCGACCACCGAGATCTACAC**ATCTTAGT**ACACTCTTTCCCTACACGAC
8	AATGATACGGCGACCACCGAGATCTACAC**GCTCCGAC**ACACTCTTTCCCTACACGAC
9	AATGATACGGCGACCACCGAGATCTACAC**CGCGGCTA**ACACTCTTTCCCTACACGAC
10	AATGATACGGCGACCACCGAGATCTACAC**TTATTCGT**ACACTCTTTCCCTACACGAC
11	AATGATACGGCGACCACCGAGATCTACAC**CCTACGAA**ACACTCTTTCCCTACACGAC
12	AATGATACGGCGACCACCGAGATCTACAC**AGCAGATC**ACACTCTTTCCCTACACGAC
13	AATGATACGGCGACCACCGAGATCTACAC**AGGAAGTC**ACACTCTTTCCCTACACGAC
14	AATGATACGGCGACCACCGAGATCTACAC**TACTTACT**ACACTCTTTCCCTACACGAC
15	AATGATACGGCGACCACCGAGATCTACAC**GCGGAGCG**ACACTCTTTCCCTACACGAC
16	AATGATACGGCGACCACCGAGATCTACAC**GCGCCTCT**ACACTCTTTCCCTACACGAC

**PCR2-R**	**First half of non-bold sequences are adapters P7, bold sequences are indexes i7, second half of non-bold sequences are partial RD2SP**
1	CAAGCAGAAGACGGCATACGAGAT**ATGCGGCT**GTGACTGGAGTTCAGACGTG
2	CAAGCAGAAGACGGCATACGAGAT**GCCTCTCT**GTGACTGGAGTTCAGACGTG
3	CAAGCAGAAGACGGCATACGAGAT**GCCGTAGG**GTGACTGGAGTTCAGACGTG
4	CAAGCAGAAGACGGCATACGAGAT**GGTCACGA**GTGACTGGAGTTCAGACGTG
5	CAAGCAGAAGACGGCATACGAGAT**ACAGTGGT**GTGACTGGAGTTCAGACGTG
6	CAAGCAGAAGACGGCATACGAGAT**CAGATCCA**GTGACTGGAGTTCAGACGTG
7	CAAGCAGAAGACGGCATACGAGAT**GTGAATAT**GTGACTGGAGTTCAGACGTG
8	CAAGCAGAAGACGGCATACGAGAT**CATAGAGT**GTGACTGGAGTTCAGACGTG
9	CAAGCAGAAGACGGCATACGAGAT**TTATAACC**GTGACTGGAGTTCAGACGTG
10	CAAGCAGAAGACGGCATACGAGAT**GGACTTGG**GTGACTGGAGTTCAGACGTG
11	CAAGCAGAAGACGGCATACGAGAT**ATCCACTG**GTGACTGGAGTTCAGACGTG
12	CAAGCAGAAGACGGCATACGAGAT**CAAGCTAG**GTGACTGGAGTTCAGACGTG
13	CAAGCAGAAGACGGCATACGAGAT**CTGCTTCC**GTGACTGGAGTTCAGACGTG
14	CAAGCAGAAGACGGCATACGAGAT**CCAAGTCT**GTGACTGGAGTTCAGACGTG
15	CAAGCAGAAGACGGCATACGAGAT**ACTGCTTA**GTGACTGGAGTTCAGACGTG

Table S3 Alpha diversity indicated by observed features, evenness and shannon index among all samples.

Groups	Sample name	Observed_features	Evenness	Shannon index
Flower buds	Flower buds1	74	0.677800575	4.208771063
	Flower buds2	81	0.651743629	4.131956846
	Flower buds3	86	0.714507475	4.591614201
	Flower buds4	90	0.619965963	4.024727958
	Flower buds5	61	0.698545253	4.142888416
	Flower buds6	95	0.643758812	4.229402443
	Flower buds7	67	0.676073047	4.101119404
Young pods	Young pods1	81	0.814458473	5.163544555
	Young pods2	87	0.773918216	4.986311335
	Young pods3	105	0.732154004	4.91586174
	Young pods4	58	0.700579994	4.10398429
	Young pods5	136	0.794678935	5.632257423
	Young pods6	162	0.76897906	5.644190958
	Young pods7	117	0.783035731	5.379741063
20daf seeds	20daf seeds1	258	0.615745396	4.932876295
	20daf seeds2	242	0.699489544	5.539162034
	20daf seeds3	298	0.698196758	5.738596818
	20daf seeds4	155	0.811035608	5.901195983
	20daf seeds5	191	0.711868235	5.394130884
	20daf seeds6	254	0.777851801	6.214012773
	20daf seeds7	162	0.619254663	4.545236338
	20daf seeds8	172	0.69898981	5.190883391
30daf seeds	30daf seeds1	206	0.770792543	5.924697292
	30daf seeds2	158	0.737952357	5.389842216
	30daf seeds3	131	0.793044146	5.577814936
	30daf seeds4	128	0.62123878	4.348671459
	30daf seeds5	137	0.730557685	5.185521886
	30daf seeds6	241	0.689885562	5.458988104
40daf seeds	40daf seeds1	271	0.618930016	5.002284639
	40daf seeds2	274	0.760643975	6.159719312
	40daf seeds3	193	0.860197352	6.531011442
	40daf seeds4	243	0.732151141	5.802160516
	40daf seeds5	163	0.717614496	5.273553848
	40daf seeds6	224	0.794511404	6.203032521
	40daf seeds7	333	0.746045333	6.251396124
	40daf seeds8	394	0.842233492	7.26178081

Groups	Sample name	Observed_features	Evenness	Shannon index
50daf seeds	50daf seeds1	223	0.859993892	6.708726263
	50daf seeds2	130	0.839525557	5.895457247
	50daf seeds3	235	0.799023791	6.29352443
	50daf seeds4	263	0.875269343	7.036219341
	50daf seeds5	96	0.841171849	5.539085081
	50daf seeds6	299	0.878633277	7.225881541
	50daf seeds7	218	0.868905744	6.749819982
Parental seeds	Parental seeds1	523	0.784946237	7.088588189
	Parental seeds2	222	0.13472536	1.050105486
	Parental seeds3	119	0.108934419	0.75108297
	Parental seeds4	260	0.203567695	1.633094927
	Parental seeds5	204	0.304043035	2.332747489
	Parental seeds6	291	0.266102245	2.178013704
Mature seeds	Mature seeds1	367	0.787940448	6.712966009
	Mature seeds2	369	0.378592138	3.228435752
	Mature seeds3	136	0.256623013	1.818806072
	Mature seeds4	244	0.201063004	1.594577871
	Mature seeds5	172	0.157306667	1.168200959
	Mature seeds6	328	0.569397799	4.758771717
negative control	YX1	1		
	YX2	5		
	YX3	11		
	YX4	0		
	YX5	5		
	YX6	5		

Table S4 Beta diversity among all groups. Significance of the microbial community dissimilarities among different groups was based on PERMANOVA tests.

Groups		*P*-value	Groups		*P*-value
Flower buds	Young pods	0.0007999	20daf seeds	40daf seeds	0.0002
Flower buds	20daf seeds	0.0005	20daf seeds	50daf seeds	0.0005
Flower buds	30daf seeds	1.00E-04	20daf seeds	Mature seeds	0.00069993
Flower buds	40daf seeds	0.0003	20daf seeds	Parental seeds	0.00029997
Flower buds	50daf seeds	0.0007999	30daf seeds	40daf seeds	0.0004
Flower buds	Mature seeds	0.00079992	30daf seeds	50daf seeds	0.0008999
Flower buds	Parental seeds	0.00059994	30daf seeds	Mature seeds	0.00159984
Young pods	20daf seeds	0.0028997	30daf seeds	Parental seeds	0.00169983
Young pods	30daf seeds	0.0012999	40daf seeds	50daf seeds	0.0011999
Young pods	40daf seeds	0.0003	40daf seeds	Mature seeds	0.00039996
Young pods	50daf seeds	0.0006999	40daf seeds	Parental seeds	0.00029997
Young pods	Mature seeds	0.0009999	50daf seeds	Parental seeds	0.00079992
Young pods	Parental seeds	0.00089991	50daf seeds	Mature seeds	0.00079992
20daf seeds	30daf seeds	0.0004	Parental seeds	Mature seeds	0.5941406

Table S5 The taxa with relative abundance greater than 1% of seed microbiota at the phylum level.

	Parental seeds	Mature seeds	Flower buds	Young pods	20daf seeds	30daf seeds	40daf seeds	50daf seeds
Gammaproteobacteria	84.04	78.77	23.91	30.64	32.05	34.12	23.80	24.72
Actinobacteriota	3.82	4.38	34.51	26.98	25.11	24.93	21.44	28.53
Firmicutes	1.72	2.76	13.49	13.61	21.24	28.79	34.66	22.13
Alphaproteobacteria	8.66	11.72	8.08	12.33	7.14	7.01	14.73	17.74
Bacteroidota	0.52	0.98	15.90	11.39	11.29	4.10	3.40	4.43
Fusobacteriota	0.30	0.25	2.72	1.99	1.92	0.54	0.71	0.77
Others	0.93	1.13	1.39	3.05	1.25	0.50	1.26	1.67

Differential taxa of seed microbiota at the phylum level.

**Phylum**	**Enrich in Groups (LDA=2)**
Un Proteobacteria	Parental seeds
Gammaproteobacteria	Parental seeds
Alphaproteobacteria	50daf seeds
Bdellovibrionota	40daf seeds
Firmicutes	40daf seeds
Fusobacteriota	Flower buds
Bacteroidota	Flower buds
Actinobacteriota	Flower buds

Table S6 Top 10 genera in relative abundance (%) of each group.

Genus	Flower buds	Genus	Young pods	Genus	20daf seeds
Actinomyces	28.12	Actinomyces	20.08	Actinomyces	16.92
Neisseria	10.27	Streptococcus	8.35	Streptococcus	13.49
Streptococcus	9.09	Brevundimonas	6.77	Neisseria	8.43
Prevotella	7.23	Haemophilus	6.23	Rhodanobacteraceae	4.97
Haemophilus	5.35	Pseudomonas	5.94	Haemophilus	4.96
Alloprevotella	4.63	Prevotella	5.09	Lautropia	4.65
Brevundimonas	3.32	Alloprevotella	4.85	Alloprevotella	4.08
Capnocytophaga	3.08	Neisseria	4.58	Prevotella	3.59
Granulicatella	2.49	Corynebacterium	3.06	Brevundimonas	3.42
Corynebacterium	1.82	Staphylococcus	2.16	Rothia	2.38
Others	24.60	Others	32.90	Others	33.10
**Genus**	**30daf seeds**	**Genus**	**40daf seeds**	**Genus**	**50daf seeds**
Staphylococcus	19.37	Bacillus	14.12	Staphylococcus	10.98
Corynebacterium	14.59	Staphylococcus	10.24	Corynebacterium	8.75
Alicycliphilus	7.68	Corynebacterium	7.53	Actinomyces	5.77
Pseudomonas	6.65	Rhodanobacteraceae	5.01	Streptococcus	5.59
Ralstonia	5.91	Allorhizobium	4.82	Sphingomonas	3.68
Actinomyces	5.29	Streptococcus	3.72	Comamonadaceae	3.62
Streptococcus	3.65	Comamonadaceae	3.58	Brevundimonas	3.38
Limnobacter	1.95	Actinomyces	2.30	Alicycliphilus	2.69
Anaerococcus	1.78	Rhodococcus	2.18	Neisseria	2.48
Neisseria	1.72	Paenibacillus	1.82	Massilia	2.23
Others	31.41	Others	44.68	Others	50.83
**Genus**	**Parental seeds**	**Genus**	**Mature seeds**		
Ralstonia	71.98	Ralstonia	61.99		
Delftia	3.05	Bosea	3.12		
Bosea	2.57	Pseudomonas	2.48		
Ochrobactrum	1.92	Comamonadaceae	1.85		
Comamonadaceae	1.70	Caulobacteraceae2	1.80		
Pseudomonas	1.11	Actinomyces	1.60		
Knoellia	1.01	Ochrobactrum	1.59		
Oxalobacteraceae	0.88	Delftia	1.47		
Actinomyces	0.88	Pantoea	1.17		
Caulobacteraceae2	0.81	Erwinia	1.00		
Others	14.09	Others	21.93		

Differential taxa of seed microbiota at the genus level.

thresholds (LDA=4)	Enrich in Groups
Delftia	Parental seeds
Proteobacteria	Parental seeds
Ochrobactrum	Parental seeds
Ralstonia	Parental seeds
Sphingomonas	50daf seeds
Comamonadaceae	50daf seeds
Rhodococcus	40daf seeds
Rhodanobacteraceae	40daf seeds
Bacillus	40daf seeds
Allorhizobium_Neorhizobium_Pararhizobium_Rhizobium	40daf seeds
Alicycliphilus	30daf seeds
Staphylococcus	30daf seeds
Corynebacterium	30daf seeds
Streptococcus	20daf seeds
Rothia	20daf seeds
Lautropia	20daf seeds
Alloprevotella	Young pods
Brevundimonas	Young pods
Microbacterium	Young pods
Haemophilus	Young pods
Capnocytophaga	Flower buds
Actinomyces	Flower buds
Neisseria	Flower buds
Prevotella	Flower buds
Granulicatella	Flower buds

Table S7 The top 20 functions with abundance relative of seed microbiota at second level.

KEGG pathways (%)	Flower buds	Young pods	20daf seeds	30daf seeds	40daf seeds	50daf seeds
Amino acid metabolism	10.29	10.53	10.39	10.96	11.06	11.09
Metabolism of cofactors and vitamins	10.43	9.70	10.17	9.58	9.66	9.31
Carbohydrate metabolism	9.95	9.59	9.71	9.51	9.72	9.59
Metabolism of other amino acids	7.71	7.33	7.59	7.39	7.16	7.19
Lipid metabolism	4.73	5.25	4.99	5.84	6.11	6.10
Global and overview maps	5.51	5.45	5.49	5.56	5.50	5.50
Biosynthesis of other secondary metabolites	5.59	5.31	5.57	4.81	5.00	4.98
Xenobiotics biodegradation and metabolism	3.46	5.02	3.87	5.61	5.58	6.02
Replication and repair	5.44	4.76	5.17	4.46	4.37	4.37
Energy metabolism	4.43	4.18	4.28	4.12	4.01	3.98
Glycan biosynthesis and metabolism	4.36	3.50	3.94	2.80	2.86	2.85
Metabolism of terpenoids and polyketides	2.82	3.20	2.86	3.44	3.50	3.57
Folding sorting and degradation	2.98	2.76	2.92	2.85	2.75	2.65
Translation	3.06	2.61	2.92	2.40	2.31	2.34
Cell motility	1.32	2.32	1.93	2.77	2.97	2.51
Cell growth and death	2.17	2.08	1.96	1.88	1.85	1.96
Membrane transport	1.74	1.87	1.82	1.92	1.75	1.68
Drug resistance: antimicrobial	1.82	1.74	1.85	1.67	1.58	1.54
Cellular community - prokaryotes	1.30	1.62	1.51	1.70	1.56	1.48
Nucleotide metabolism	1.65	1.46	1.57	1.35	1.32	1.31
Others	9.23	9.74	9.48	9.38	9.39	9.98

Table S8 The top 40 functions with abundance relative of seed microbiota at third level.

KEGG Level 3 (%)	Flower buds	Young pods	20daf seeds	30daf seeds	40daf seeds	50daf seeds
Valine leucine and isoleucine biosynthesis	2.13	2.10	2.16	2.04	1.96	2.06
D-Alanine metabolism	2.18	1.96	2.18	2.05	1.83	1.81
Lipoic acid metabolism	2.06	1.64	1.86	1.66	1.73	1.61
D-Glutamine and D-glutamate metabolism	1.99	1.70	1.84	1.60	1.52	1.57
Synthesis and degradation of ketone bodies	0.97	1.41	1.19	1.90	1.93	2.05
Fatty acid biosynthesis	1.54	1.46	1.54	1.42	1.50	1.40
Biosynthesis of amino acids	1.46	1.35	1.43	1.34	1.30	1.30
Biotin metabolism	1.30	1.37	1.41	1.36	1.42	1.31
Cell cycle - Caulobacter	1.51	1.43	1.39	1.23	1.28	1.31
Biosynthesis of terpenoids and steroids	1.09	1.53	1.34	1.08	1.35	1.68
Mismatch repair	1.54	1.33	1.45	1.25	1.18	1.18
Bacterial chemotaxis	0.77	1.33	1.06	1.50	1.64	1.39
Aminoacyl-tRNA biosynthesis	1.49	1.28	1.43	1.19	1.15	1.16
Protein export	1.40	1.24	1.37	1.24	1.23	1.19
Peptidoglycan biosynthesis	1.43	1.25	1.42	1.20	1.24	1.14
Streptomycin biosynthesis	1.50	1.32	1.42	1.10	1.12	1.16
Pantothenate and CoA biosynthesis	1.34	1.24	1.31	1.21	1.20	1.21
Ribosome	1.46	1.24	1.39	1.13	1.08	1.10
One carbon pool by folate	1.33	1.21	1.27	1.07	1.03	1.07
Citrate cycle (TCA cycle)	1.15	1.07	1.10	1.14	1.11	1.10
Carbon fixation in photosynthetic organisms	1.27	1.13	1.21	1.00	1.00	1.02
Drug metabolism - other enzymes	1.17	1.11	1.15	1.00	1.01	1.04
Selenocompound metabolism	1.07	1.01	1.08	0.97	1.05	0.98
Flagellar assembly	0.55	0.99	0.87	1.28	1.32	1.12
Alanine aspartate and glutamate metabolism	1.08	0.98	1.04	0.94	0.98	0.95
Pyruvate metabolism	0.98	0.95	0.97	1.04	1.01	1.00
Homologous recombination	1.16	0.98	1.10	0.90	0.85	0.86
Fatty acid metabolism	0.88	0.94	0.91	1.00	1.04	1.01
Thiamine metabolism	0.95	0.91	0.98	1.00	1.01	0.91
Lysine biosynthesis	1.08	0.95	1.04	0.91	0.88	0.89
Sulfur relay system	0.90	0.91	0.89	1.05	0.96	0.91
Vancomycin resistance	1.09	0.94	1.04	0.86	0.84	0.83
Glycolysis / Gluconeogenesis	1.00	0.89	0.96	0.90	0.91	0.89
Bacterial secretion system	0.87	1.00	0.90	1.01	0.87	0.90
Glycine serine and threonine metabolism	0.93	0.90	0.91	0.93	0.90	0.90
Phenylalanine tyrosine and tryptophan biosynthesis	0.99	0.89	0.96	0.84	0.80	0.81
DNA replication	0.99	0.86	0.94	0.83	0.78	0.79
C5-Branched dibasic acid metabolism	0.91	0.89	0.89	0.83	0.80	0.84
Base excision repair	0.89	0.83	0.89	0.81	0.82	0.81
Glyoxylate and dicarboxylate metabolism	0.70	0.79	0.72	0.89	0.90	0.91
Others	50.92	52.69	51.00	53.29	53.46	53.84

Table S9 The composition and relative abundance of 61 core microbiota. The red part was the relative abundance of core ASV S1.

Genus (%)	Parental seeds	Mature seeds	Flower buds	Young pods	20daf seeds	30daf seeds	40daf seeds	50daf seeds
Ralstonia	70.816	61.219	1.122	1.671	0.323	5.805	0.212	0.271
Actinomyces1	0.654	0.987	24.163	12.158	11.495	3.223	1.355	3.566
Staphylococcus1	0.192	0.294	0.425	0.503	0.549	11.179	5.286	4.175
Neisseria1	0.211	0.243	6.715	2.964	4.776	1.336	0.825	1.836
Streptococcus1	0.213	0.113	4.958	2.590	4.519	1.445	0.989	1.914
Brevundimonas	0.400	0.666	2.989	6.482	2.848	0.480	0.890	0.758
Haemophilus1	0.151	0.216	2.989	5.306	3.752	1.418	0.313	0.819
Actinomyces2	0.123	0.232	2.082	4.844	2.751	0.929	0.305	0.687
Alicycliphilus	0.070	0.102	0.017	0.015	0.019	7.439	1.118	2.607
Staphylococcus2	0.208	0.147	0.169	0.692	0.675	3.290	1.255	2.984
Alloprevotella1	0.028	0.049	2.484	1.937	1.512	0.596	0.316	0.192
Streptococcus2	0.118	0.169	1.106	0.675	2.760	0.849	0.691	0.717
Allorhizobium	0.030	0.104	0.849	0.021	0.242	0.004	4.366	0.356
Lautropia	0.033	0.078	0.251	0.371	4.654	0.152	0.078	0.306
Actinomyces3	0.018	0.005	1.593	1.879	1.232	0.476	0.390	0.291
Prevotella1	0.049	0.011	2.413	1.290	0.884	0.183	0.002	0.743
Corynebacterium1	0.089	0.021	0.059	0.070	0.187	3.112	1.047	0.856
Streptococcus3	0.053	0.063	0.873	1.917	0.881	0.172	0.149	1.192
Novosphingobium	0.147	0.008	0.716	1.337	0.255	0.040	0.527	1.953
Neisseria2	0.108	0.064	1.505	1.021	1.591	0.129	0.069	0.166
Granulicatella1	0.091	0.024	1.675	0.835	0.986	0.506	0.004	0.191
Haemophilus2	0.017	0.011	2.352	0.861	0.695	0.090	0.139	0.144
Fusobacterium	0.192	0.183	1.154	1.167	0.678	0.141	0.436	0.119
Escherichia-Shigella	0.003	0.062	0.855	0.668	0.189	0.024	1.584	0.408
Capnocytophaga	0.034	0.021	1.274	0.678	0.811	0.601	0.027	0.305
Prevotella2	0.034	0.013	2.206	0.331	0.705	0.117	0.048	0.022
Streptococcus4	0.012	0.051	0.560	0.975	0.974	0.407	0.081	0.402
Alloprevotella2	0.003	0.054	1.042	1.239	0.730	0.333	0.010	0.003
Ochrobactrum	1.646	1.390	0.020	0.009	0.001	0.010	0.009	0.048
Enterobacteriaceae	0.252	0.018	0.179	0.803	0.760	0.577	0.402	0.082
Streptococcus5	0.007	0.027	0.982	0.597	0.435	0.064	0.250	0.464
Streptococcus6	0.050	0.204	0.031	0.348	1.283	0.289	0.182	0.380
Alloprevotella3	0.022	0.011	0.656	1.080	0.731	0.133	0.015	0.096
Comamonadaceae1	0.068	0.062	0.167	0.048	0.202	0.141	1.230	0.683
Corynebacterium2	0.006	0.013	0.469	1.166	0.530	0.142	0.156	0.046
Microbacterium	0.020	0.034	0.262	0.801	0.060	0.195	0.437	0.660
Streptococcus7	0.013	0.007	0.013	0.020	1.975	0.018	0.238	0.014
Bosea1	0.171	0.084	0.406	0.217	0.079	0.496	0.222	0.476
Sphingomonas1	0.237	0.301	0.357	0.322	0.041	0.133	0.457	0.280
Kingella	0.008	0.020	0.630	0.696	0.513	0.137	0.054	0.029

Genus (%)	Parental seeds	Mature seeds	Flower buds	Young pods	20daf seeds	30daf seeds	40daf seeds	50daf seeds
Leptotrichia	0.080	0.064	0.428	0.616	0.413	0.154	0.023	0.079
Granulicatella2	0.001	0.005	0.458	1.036	0.136	0.030	0.091	0.065
Janibacter	0.026	0.046	0.165	0.040	0.077	0.316	0.610	0.372
Bosea2	0.008	0.037	0.070	0.708	0.091	0.068	0.061	0.338
Comamonadaceae2	0.008	0.021	0.070	0.025	0.007	0.170	0.859	0.183
Staphylococcus3	0.068	0.068	0.294	0.215	0.056	0.038	0.077	0.379
Diaphorobacter	0.003	0.118	0.030	0.001	0.016	0.571	0.132	0.225
Streptococcus8	0.002	0.019	0.002	0.887	0.150	0.002	0.011	0.009
Streptococcus9	0.004	0.034	0.199	0.012	0.173	0.020	0.450	0.102
Bacillus	0.044	0.031	0.006	0.037	0.252	0.274	0.294	0.033
Stenotrophomonas	0.056	0.375	0.005	0.213	0.006	0.209	0.039	0.063
Sphingomonas2	0.005	0.051	0.040	0.462	0.025	0.075	0.052	0.199
Phyllobacterium	0.098	0.111	0.088	0.311	0.001	0.087	0.004	0.142
Aerococcus	0.003	0.011	0.024	0.034	0.332	0.026	0.152	0.232
Rothia	0.003	0.011	0.289	0.107	0.194	0.009	0.125	0.040
Prevotella3	0.003	0.008	0.007	0.309	0.324	0.086	0.001	0.038
Corynebacterium3	0.006	0.004	0.043	0.022	0.004	0.378	0.011	0.228
Kytococcus	0.004	0.019	0.007	0.003	0.043	0.056	0.331	0.196
Cutibacterium	0.004	0.020	0.016	0.005	0.028	0.018	0.149	0.223
Chryseobacterium	0.019	0.003	0.040	0.019	0.010	0.043	0.197	0.117
Acidisoma	0.044	0.030	0.012	0.024	0.031	0.010	0.132	0.110

Table S10 The composition and relative abundance of 28 missing core microbiota.

**Relative abundance(%)**	Flower buds	Young pods	20daf seeds	30daf seeds	40daf seeds	50daf seeds
Corynebacterium2	0.961	0.758	0.494	0.165	0.018	0.068
Microbacterium	0.287	0.751	0.539	0.138	0.051	0.224
Corynebacterium3	0.067	0.065	0.030	1.302	0.292	0.179
Actinomyces3	0.193	0.354	0.591	0.376	0.031	0.248
Alloprevotella	0.265	0.454	0.749	0.158	0.004	0.085
Enterobacteriaceae	0.149	0.627	0.230	0.039	0.217	0.444
Corynebacteriaceae	0.033	0.028	0.055	0.074	0.700	0.616
Capnocytophaga	0.972	0.068	0.339	0.059	0.011	0.045
Pasteurellaceae	0.458	0.448	0.291	0.162	0.004	0.050
Lawsonella	0.058	0.111	0.131	0.094	0.251	0.478
Streptococcus	0.339	0.242	0.223	0.095	0.066	0.038
Delftia	0.028	0.210	0.083	0.350	0.120	0.135
Cloacibacterium	0.009	0.035	0.004	0.500	0.158	0.198
Leptotrichia	0.526	0.123	0.077	0.078	0.012	0.059
Corynebacterium4	0.003	0.025	0.005	0.085	0.202	0.543
Rothia	0.002	0.055	0.063	0.080	0.048	0.595
Cutibacterium	0.007	0.030	0.033	0.013	0.212	0.394
Jeotgalicoccus	0.020	0.009	0.061	0.001	0.038	0.547
Corynebacterium1	0.123	0.397	0.002	0.005	0.016	0.093
Afipia	0.002	0.007	0.028	0.089	0.060	0.417
Massilia	0.008	0.007	0.032	0.028	0.128	0.397
Comamonadaceae	0.019	0.024	0.002	0.108	0.232	0.189
Actinomyces2	0.004	0.070	0.239	0.078	0.025	0.127
Escherichia-Shigella	0.023	0.056	0.007	0.012	0.160	0.089
Atopostipes	0.023	0.007	0.066	0.011	0.081	0.125
Pseudomonas	0.020	0.022	0.008	0.036	0.040	0.173
Haemophilus	0.010	0.002	0.039	0.155	0.038	0.002
Actinomyces1	0.005	0.129	0.019	0.059	0.004	0.021

Table S11 The number of reads of ASVs among the samples of negative control and the ASVs labeled in red were overlapped with core microbiota.

ASVs in negative control	Sample1	Sample2	Sample3	Sample4	Sample5	Sample6
Actinomyces	0	0	22	0	12	26
Cellvibrio	0	0	43	0	0	0
Pseudomonas	0	15	0	0	0	0
Arenimonas	0	0	14	0	0	0
Anaerococcus	0	13	0	0	0	0
Corynebacterium	0	0	12	0	0	0
Comamonadaceae	0	10	0	0	0	0
Xanthomonadaceae	0	10	0	0	0	0
Bacteria	9	0	0	0	0	0
Massilia	0	0	9	0	0	0
Corynebacterium	0	0	0	0	0	8
uncultured Neisseriaceae	0	0	7	0	0	0
Sordariomycetes	0	0	0	0	0	6
Prevotella	0	0	6	0	0	0
Neisseria	0	0	0	0	0	5
Corynebacterium	0	5	0	0	0	0
Ralstonia	0	0	5	0	0	0
Faecalibacterium	0	0	5	0	0	0
Haemophilus	0	0	0	0	4	0
Neisseria	0	0	0	0	4	0
Stenotrophomonas	0	0	4	0	0	0
Neisseria	0	0	4	0	0	0
Ralstonia	0	0	0	0	4	0
Prevotella	0	0	0	0	3	0
Leptotrichia	0	0	0	0	0	2

Table S12 16S rRNA gene sequence of Core ASV S1, *Sp. endophytica* and *Ralstonia pickettii*

>the 16S rRNA gene of core ASV *Sphingomonas*
GTAGTCCACGCCGTAAACGATGATAACTAGCTGTCCGGGCACTTGGTGCTTGGGTGGC
GCAGCTAACGCATTAAGTTATCCGCCTGGGGAGTACGGTCGCAAGATTAAAACTCAAA
GGAATTGACGGGGGCCTGCACAAGCGGTGGAGCATGTGGTTTAATTCGAAGCAACGC
GCAGAACCTTACCAGCGTTTGACATGTCCGGACGATTTCCAGAGATGGATCTCTTCCCT
TCGGGGACTGGAACACAGGTGCTGCATGGCTGTCGTCAGCTCGTGTCGTGAGATGTTG
GGTTAAGTCCCGCAACGAGCGCAACCCTCGCCTTTAGTTACCATCATTTAGTTGGGGAC
TCTAAAGGAACCGCCGGTGATAAGCCGGA

>the 16S rRNA gene of strain *Sphingomonas endophytica*
GCAAGTCGAACGATGCTTTCGGGCATAGTGGCGCACGGGTGCGTAACGCGTGGGAATC
TGCCCTTGGGTCTGGGATAACAGTTGGAAACGACTGCTAATACCGGATGATATCGCGAG
ATCAAAGATTTATCGCCCGAGGATGAGCCCGCGTAGGATTAGCTAGTTGGTGGGGTAAA
GGCCTACCAAGGCGACGATCCTTAGCTGGTCTGAGAGGATGATCAGCCACACTGGGAC
TGAGACACGGCCCAGACTCCTACGGGAGGCAGCAGTGGGGAATATTGGACAATGGGC
GAAAGCCTGATCCAGCAATGCCGCGTGAGTGATGAAGGCCTTAGGGTTGTAAAGCTCT
TTTACCCGGGATGATAATGACAGTACCGGGAGAATAAGCCCCGGCTAACTCCGTGCCA
GCAGCCGCGGTAATACGGAGGGGGCTAGCGTTGTTCGGAATTACTGGGCGTAAAGCGC
ACGTAGGCGGCTTTGTAAGTCAGGGGTGAAAGCCTGGAGCTCAACTCCAGAACTGCC
TTTGAGACTGCATCGCTTGAATCCGGGAGAGGTAAGTGGAATTCCGAGTGTAGAGGTG
AAATTCGTAGATATTCGGAAGAACACCAGTGGCGAAGGCGGCTTACTGGACCGGGATT
GACGCTGAGGTGCGAAAGCGTGGGGAGCAAACAGGATTAGATACCCTGGTAGTCCAC
GCCGTAAACGATGATAACTAGCTGTCCGGGGACTTGGTCTTTGGGTGGCGCAGCTAAC
GCATTAAGTTATCCGCCTGGGGAGTACGGCCGCAAGGTTAAAACTCAAAGGAATTGAC
GGGGGCCTGCACAAGCGGTGGAGCATGTGGTTTAATTCGAAGCAACGCGCAGAACCT
TACCAGCGTTTGACATGTCCGGACGATTTCCAGAGATGGATCTCTTCCCTTCGGGGACT
GGAACACAGGTGCTGCATGGCTGTCGTCAGCTCGTGTCGTGAGATGTTGGGTTAAGTC
CCGCAACGAGCGCAACCCTCGCCTTTAGTTACCATCATTTAGTTGGGTACTCTAAAGGA
ACCGCCGGTGATAAGCCGGAGGAAGGTGGGGATGACGTCAAGTCCTCATGGCCCTTAC
GCGCTGGGCTACACACGTGCTACAATGGCGACTACAGTGGGCTGCAATCCCGCGAGGG
TGAGCTAATCTCCAAAAGTCGTCTCAGTTCGGATTGTTCTCTGCAACTCGAGAGCATGA
AGGCGGAATCGCTAGTAATCGCGGATCAGCATGCCGCGGTGAATACGTTCCCAGGCCT
TGTACACACCGCCCGTCACACCATGGGAGTTGGGTTCACCCGAAGGCGTTGCGCTAAC
CCGTAAGGGAGGCAG

>the 16S rRNA gene of strain *Ralstonia pickettii*
GCAAGTCGAACGGCAGCATGATCTAGCTTGCTAGATTGATGGCGAGTGGCGAACGGGT
GAGTAATACATCGGAACGTGCCCTGTAGTGGGGGATAACTAGTCGAAAGATTAGCTAAT
ACCGCATACGACCTGAGGGTGAAAGTGGGGGACCGCAAGGCCTCATGCTATAGGAGC
GGCCGATGTCTGATTAGCTAGTTGGTGGGGTAAAGGCCCACCAAGGCGACGATCAGTA
GCTGGTCTGAGAGGACGATCAGCCACACTGGGACTGAGACACGGCCCAGACTCCTAC
GGGAGGCAGCAGTGGGGAATTTTGGACAATGGGCGAAAGCCTGATCCAGCAATGCCG

CGTGTGTGAAGAAGGCCTTCGGGTTGTAAAGCACTTTTGTCCGGAAAGAAATGGCTCT
GGTTAATACCTGGGGTCGATGACGGTACCGGAAGAATAAGGACCGGCTAACTACGTGC
CAGCAGCCGCGGTAATACGTAGGGTCCAAGCGTTAATCGGAATTACTGGGCGTAAAGC
GTGCGCAGGCGGTTGTGCAAGACCGATGTGAAATCCCCGAGCTTAACTTGGGAATTGC
ATTGGTGACTGCACGGCTAGAGTGTGTCAGAGGGGGGTAGAATTCCACGTGTAGCAGT
GAAATGCGTAGAGATGTGGAGGAATACCGATGGCGAAGGCAGCCCCCTGGGATAACAC
TGACGCTCATGCACGAAAGCGTGGGGAGCAAACAGGATTAGATACCCTGGTAGTCCAC
GCCCTAAACGATGTCAACTAGTTGTTGGGGATTCATTTCCTTAGTAACGTAGCTAACGC
GTGAAGTTGACCGCCTGGGGAGTACGGTCGCAAGATTAAAACTCAAAGGAATTGACG
GGGACCCGCACAAGCGGTGGATGATGTGGATTAATTCGATGCAACGCGAAAAACCTTA
CCTACCCTTGACATGCCACTAACGAAGCAGAGATGCATTAGGTGCTCGAAAGAGAAAG
TGGACACAGGTGCTGCATGGCTGTCGTCAGCTCGTGTCGTGAGATGTTGGGTTAAGTC
CCGCAACGAGCGCAACCCTTGTCTCTAGTTGCTACGAAAGGGCACTCTAGAGAGACTG
CCGGTGACAAACCGGAGGAAGGTGGGGATGACGTCAAGTCCTCATGGCCCTTATGGGT
AGGGCTTCACACGTCATACAATGGTGCATACAGAGGGTTGCCAAGCCGCGAGGTGGA
GCTAATCCCAGAAAATGCATCGTAGTCCGGATCGTAGTCTGCAACTCGACTACGTGAA
GCTGGAATCGCTAGTAATCGCGGATCAGCATGCCGCGGTGAATACGTTCCCGGGTCTTG
TACACACCGCCCGTCACACCATGGGAGTGGGCTTTACCAGAAGTAGTTAGCCTAACCG
CAAGGAGGGC

The sequence information used to make the evolutionary tree in this research.

The name of 16S rRNA gene sequence in NCBI	GenBank
Sphingomonas yunnanensis strain 21	MN737131.1
Sphingomonas yunnanensis strain SS1-22	KU341396.1
Sphingomonas ginsenosidimutans strain P5-A12	MK318623.1
Sphingomonas ginsenosidimutans strain 20ABL7	MH482324.1
Sphingomonas adhaesiva JCM 7370	LC504024.1
Sphingomonas adhaesiva strain DSM 7418	KY927401.1
Sphingomonas melonis strain IITA-TZ131	OM909380.1
Sphingomonas melonis strain 65_5a	OP811591.1
Sphingomonas koreensis strain 234-LR45	MF077172.1
Sphingomonas koreensis strain RS8B	MG547700.1
Sphingomonas endophytica strain -Y72	JX134635.1
Sphingomonas phyllosphaerae strain L6-296	JQ659373.1
Sphingomonas phyllosphaerae strain L7-510	JQ659443.1
Sphingobium indicum strain UT26S	NCBI Reference Sequence NR_102886.2
Sphingobium indicum strain NBRC 101211	NCBI Reference Sequence NR_113986.1
Sphingomonas phyllosphaerae strain L7-512	JQ659446.1
