# Supplementary material for: The Dynamic Changes of Brassica napus Seed Microbiota across the Entire Seed Life in the Field
Source: Plants (Basel). 2024 Mar 21;13(6):912. doi: 10.3390/plants13060912 (PMC10975644; doi:10.3390/plants13060912)
Supplement: Supplementary file 1 [file plants-13-00912-s001.zip › Additional file 2 Statistical analysis in R.pdf]

α diversity boxplots

```
library("ggplot2")
design = read.table("design.txt", header=T, row.names= 1, sep="\t")
alpha = read.table("alpha.txt", header=T, row.names= 1, sep="\t")
index = cbind(alpha, design[match(rownames(alpha), rownames(design)), ])
p = ggplot(index, aes(x= Groups, y= Observed_features, color= Groups))+
geom_boxplot(alpha=1, outlier.size=0, size=0.7, width=0.5, fill="transparent") +
geom_jitter( position=position_jitter(0.17), size=1, alpha=0.7)+
labs(x=" Groups ", y=" Observed_features ")
p
ggsave(paste("alpha_Observed_features.pdf", sep=""), p, width = 7, height = 3)
ggsave(paste("alpha_Observed_features.png", sep=""), p, width = 7, height = 3)
```

β diversity - PCoA

```
library(vegan)
otu <- read.delim('otu_table.txt', row.names = 1, sep = '\t')
otu <- t(otu)
bray_dis <- vegdist(otu, method = 'bray')
bray_sim <- 1 - bray_dis
bray <- as.matrix(bray_dis)
write.table(bray, 'bray-curtis_distance.txt', col.names = NA, sep = '\t', quote = FALSE)

library("ggplot2")
library("vegan")
design = read.table("design.txt", header=T, row.names= 1, sep="\t")
bray_curtis = read.table("bray-curtis_distance.txt", sep="\t", header=T, check.names=F)
idx = rownames(design) %in% colnames(bray_curtis)
sub_design = design[idx,]
bray_curtis = bray_curtis[rownames(sub_design), rownames(sub_design)] # subset and reorder distance matrix
pcoa = cmdscale(bray_curtis, k=3, eig=T)
points = as.data.frame(pcoa$points)
colnames(points) = c("x", "y", "z")
eig = pcoa$eig
points = cbind(points, sub_design[match(rownames(points), rownames(sub_design)), ])
p = ggplot(points, aes(x=x, y=y, color= Groups)) +
  geom_point(alpha=.7, size=2) +
  labs(x=paste("PCoA 1 (", format(100 * eig[1] / sum(eig), digits=4), "%)", sep=""),
       y=paste("PCoA 2 (", format(100 * eig[2] / sum(eig), digits=4), "%)", sep=""),
       title="bray_curtis PCoA")
p
ggsave("beta_pcoa_bray_curtis.pdf", p, width = 5, height = 3)
ggsave("beta_pcoa_bray_curtis.png", p, width = 5, height = 3)
```

β diversity - Adonis analysis

```
design2 = subset(sub_design, Groups %in% c("Parental seeds","Mature seeds"))
sub_dis_table = bray_curtis[rownames(design2),rownames(design2)]
sub_dis_table <- as.dist(sub_dis_table, diag = FALSE, upper = FALSE)
adonis_table = adonis(sub_dis_table~ Groups, data=design2, permutations = 10000)
adonis_pvalue = adonis_table$aov.tab$`Pr(>F)`[1]
adonis_pvalue
```

β diversity - UPGMA

```
dat <- read.delim('otu_table.txt', row.names = 1, sep = '\t', head = TRUE, check.names = FALSE)
dat <- t(dat)
group <- read.delim('group.txt', row.names = 1, sep = '\t', head = TRUE, check.names = FALSE,
stringsAsFactors = FALSE)
dis_bray <- vegan::vegdist(dat, method = 'bray')
upgma <- hclust(dis_bray, method = 'average')
upgma
plot(upgma, main = 'UPGMA\n(Bray-curtis distance)', sub = '', xlab = 'Sample', ylab = 'Height')

par(mfrow = c(1, 2))
plot(upgma, hang = -1, main = 'UPGMA\n(Bray-curtis distance)', sub = '', xlab = 'Sample', ylab =
'Height')
plot(as.dendrogram(upgma), main = 'UPGMA\n(Bray-curtis distance)', sub = '',ylab = 'Height')
```
